# Microwave-Assisted Doping Engineering Construction of Spinel-Structured Nonstoichiometric Manganese Cobaltite with Mixed 1D/2D Morphology for Supercapacitor Application

**DOI:** 10.3390/molecules30040873

**Published:** 2025-02-14

**Authors:** Yuxuan Sheng, Yin Sun, Jin Yan, Wei Wang, Shuhuang Tan, Yuchen Lin, Haowei Wang, Yichen Liu, Baotong Xie, Xiaoran Sun

**Affiliations:** 1 Naval Architecture and Shipping College, Guangdong Ocean University, Zhanjiang 524088, China; 2 Guangdong Provincial Key Laboratory of Intelligent Equipment for South China Sea Marine Ranching, Guangdong Ocean University, Zhanjiang 524088, China; 3 School of Mechanical Engineering, Guangdong Ocean University, Zhanjiang 524088, China

**Keywords:** microwave-assisted doping engineering, significant defects, Ni doping, A_1−x_B_3−x_O_4_, high specific capacitance, wide potential window, supercapacitor

## Abstract

High-performance electrode materials are fundamental to improving supercapacitor performance, serving as key factors in developing devices with high energy density, high power density, and excellent cyclic stability. Non-stoichiometric spinels with phase deficiencies can achieve electrochemical performance that surpasses that of stoichiometric materials, owing to their unique structural characteristics. In this study, we used a microwave-assisted method to synthesize a high-performance non-stoichiometric spinel material with phase deficiencies, Mn_0.5_Co_2.5_O_4_, which displayed a wide potential window (1.13 V in a traditional aqueous three-electrode system) and high specific capacitance (716.9 F g^−1^ at 1 A g^−1^). More critically, through microwave-assisted doping engineering, nickel was successfully doped into the phase-deficient Mn_0.5_Co_2.5_O_4_, resulting in a spinel material, Ni−Mn_0.5_Co_2.5_O_4_, with significant lattice defects and a mixed 1D/2D morphology. The doping of nickel effectively promoted the high-state conversion of manganese valence states within the manganese cobaltite material, substantially increasing the quantity of highly active Co^3+^ ions. These changes led to an increase in the density of reactive sites, effectively promoting synergistic interactions, thereby significantly enhancing the material’s conductivity and energy storage performance. The specific capacitance of Ni−Mn_0.5_Co_2.5_O_4_ reached 1180.6 F g^−1^ at 1 A g^−1^, a 64.7% improvement over the original Mn_0.5_Co_2.5_O_4_; at a high current density of 10 A g^−1^, the capacitance increased by 14.3%. Notably, the charge transfer resistance was reduced by a factor of 41.6. After 5000 cycles of testing, the capacity retention stood at 79.2%. This work reveals the effectiveness of microwave-assisted doping engineering in constructing high-performance non-stoichiometric spinel-type bimetallic oxide materials, offering advanced strategies for the development of high-performance electrode materials.

## 1. Introduction

With the increasing global demand for energy in recent years, supercapacitors have undergone rapid development due to their stable cycling performance, high power density, and fast charge/discharge capabilities. As the key component of supercapacitors, electrode materials play a crucial role in determining the overall performance of these devices. Spinel-type bimetallic oxides (AB_2_O_4_) have garnered widespread attention for their high power density and fast charge/discharge properties [1]. Among these materials, MnCo_2_O_4_ has demonstrated significant potential for practical applications, owing to its unique structural characteristics, high surface activity, abundant redox couples, and the ability to be effectively tailored and easily controlled in its microstructure [2,3,4]. However, the stoichiometric MnCo_2_O_4_ reported in the existing literature often presents limitations such as a narrow potential window, low conductivity, and restricted specific capacitance, which prevent it from meeting the requirements for practical supercapacitor electrode applications [5,6,7].

Non-stoichiometric AB_2_O_4_-type materials, such as A_1−x_B_3−x_O_4_, exhibit slight deviations in their stoichiometric ratios, which facilitate the enhancement of conductivity, carrier concentration, and the regulation of spatial charge distribution in bimetallic oxides. Sun et al. reported that Mn_0.65_Co_2.35_O_4_, owing to structural defects and charge imbalance between Co^3+^ and Mn^4+^, generates additional vacancies, thereby demonstrating enhanced catalytic activity [8]. Wang et al. demonstrated the synergistic effect between Mn_0.5_Co_2.5_O_4_ nanofibers and graphene layers, which effectively prevents the aggregation of Mn_0.5_Co_2.5_O_4_ nanofibers and the stacking of graphene layers, while increasing the contact area between the active materials and the electrolyte. Additionally, the graphene layers provide a good conductive pathway, reducing volume changes during the charge/discharge process [9,10].

In our previous work, we synthesized non-stoichiometric, oxygen vacancy-rich, and unique MnCo_2_O_4.5_/a-CC porous nanosheets using a microwave-assisted process. These materials exhibited a high specific capacitance of 522.7 C g^−1^ at 1 A g^−1^ and maintained 87.6% of their initial capacitance after 5000 cycles at 10 A g^−1^, demonstrating excellent cycling stability [11]. This work demonstrates that the unique microwave effects inherent in the microwave-assisted process provide a feasible pathway for the precise construction of non-stoichiometric AB_2_O_4_-type materials.

In addition, doping engineering, as a widely employed material modification strategy, has been utilized to modulate the electronic structure, enhance active adsorption and energy storage sites, improve charge transfer and separation within the material, and boost electrochemical performance, resulting in enhanced electron transfer capability [12], high conductivity [13], and superior specific capacitance [14]. Mazinani et al. doped Ag into MnCo_2_O_4_, resulting in a high specific capacitance of 942 F g^−1^ at 1 A g^−1^ and 590 F g^−1^ at 20 A g^−1^ along with excellent rate performance [15]. Kalawa et al. synthesized nickel-doped MnCo_2_O_4_ nanoparticles with ultrafine particle sizes [16]. Khamsanga demonstrated that Ni doping enhances the oxygen vacancy concentration, thereby modulating the valence states of Mn and Co, which resulted in rechargeable zinc–air batteries exhibiting an exceptional specific capacitance of 808.6 mA h g^−1^ at 1.0 mA cm^2^ [17]. Shi et al. showed that doping MnCo_2_O_4_ with Ni and Cu to form Mn_1_Co_1.9_Ni_0.1_O_4_ and Mn_1_Co_1.7_Cu_0.3_O_4_, respectively, increased the ion pair concentration, thereby enhancing the material’s conductivity [18]. Moreover, to the best of the authors’ knowledge, no studies have yet been conducted on doping non-stoichiometric AB_2_O_4_-type materials.

Herein, we synthesized a non-stoichiometric bimetallic oxide material, Mn_0.5_Co_2.5_O_4_, using a microwave-assisted process. Based on this, we successfully prepared a Ni-doped non-stoichiometric spinel material, Ni−Mn_0.5_Co_2.5_O_4_, through microwave-assisted doping engineering. Systematic characterization and analysis showed that the microwave-assisted doping had no significant effect on the lattice stability. However, after Ni doping regulation, the microstructure of the material was successfully transformed from nanowires into a mixed one-dimensional/two-dimensional nanosheet-wire hierarchical structure. Additionally, changes in the oxidation states of the elements were observed: the highest oxidation state of Mn shifted from Mn^3+^ to Mn^4+^, and the proportion of highly active Co^3+^ significantly increased, resulting in an increased number of active sites. Consequently, the conductivity and energy storage performance of the material were significantly enhanced. 

Compared to non-stoichiometric Mn_0.5_Co_2.5_O_4_, which has a specific capacitance of 716.9 F g^−1^ at 1 A g^−1^, the Ni-doped Mn_0.5_Co_2.5_O_4_ exhibited a significant 64.7% increase, reaching 1180.6 F g^−1^ at 1 A g^−1^. Additionally, the specific capacitance retention improved by 14.3% at 10 A g^−1^, and the charge transfer resistance was reduced 41.6-fold. This work demonstrates the effectiveness of microwave-assisted doping engineering in constructing high-performance spinel-type bimetallic oxide materials, thereby providing a viable strategy for the development of advanced supercapacitor electrode materials.

## 2. Results and Discussion

### 2.1. Material Testing Results

**Figure 1:**
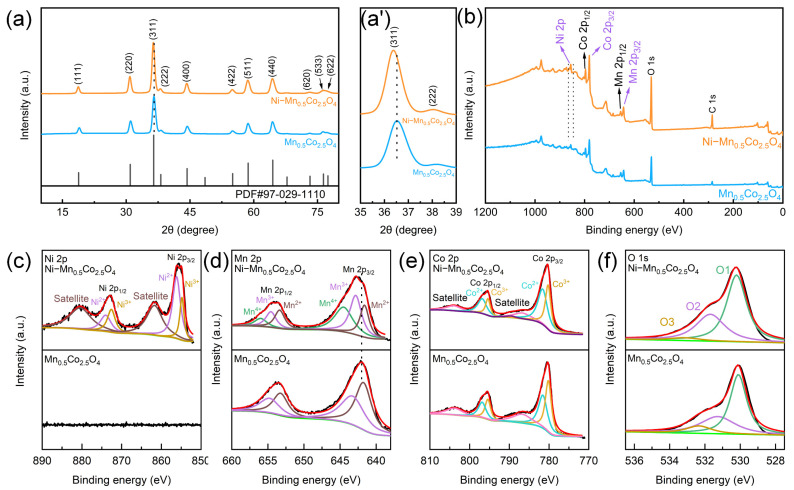
(**a**) XRD pattern and (**a′**) partial enlargement of Mn_0.5_Co_2.5_O_4_ and Ni−Mn_0.5_Co_2.5_O_4_; (**b**) XPS survey spectrum; (**c**) Ni 2p; (**d**) Mn 2p; (**e**) Co 2p; and (**f**) O1s.

Figure 1a,a′ shows the XRD patterns of the two samples, along with their locally magnified views. Figure 1a clearly shows that the XRD pattern of Mn_0.5_Co_2.5_O_4_ matches well with the standard card (PDF#97-029-1110), indicating that the pure-phase Mn_0.5_Co_2.5_O_4_ has been successfully synthesized, confirming that it adopts a cubic spinel structure (space group Fd$\bar{3}$m) [19]. In the XRD pattern of Ni−Mn_0.5_Co_2.5_O_4_, aside from a leftward shift of the (311) main peak and an increase in peak intensity, no peak splitting or formation of new peaks was observed. The unit cell parameters of both samples were calculated using Scherrer’s equation (Supporting Information Equation (S2)) and Bragg’s law (Supporting Information Equations (S3) and (S4)), as summarized in Table S1 of the Supporting Information [16]. The lattice parameters, interplanar spacings, and unit cell parameters of Ni−Mn_0.5_Co_2.5_O_4_ were slightly larger compared to those of Mn_0.5_Co_2.5_O_4_, which could be attributed to the substitution of smaller Mn^2+^ ions (0.67 Å) with larger Ni^2+^ ions (0.69 Å) [11,20,21]. This substitution may lead to stretching and deformation observed on the (311) crystal plane [22]. Additionally, from the unit cell parameters, it can be concluded that the unit cell of Mn_0.5_Co_2.5_O_4_ is still sufficiently capable of accommodating doping-induced defects, meaning that Ni doping only causes a slight lattice expansion without disrupting the original crystal structure of Mn_0.5_Co_2.5_O_4_ [23]. It is worth noting that, although no significant changes in unit cell parameters were observed after Ni doping, the doping still influenced the oxidation states of the elements and the number of active sites, as evidenced by XPS analysis.

Figure S1 shows the Raman spectra of Mn_0.5_Co_2.5_O_4_ and Ni−Mn_0.5_Co_2.5_O_4_ samples. The peak at 179.8 cm^−1^ in Mn_0.5_Co_2.5_O_4_ may be related to the low-frequency metal-oxygen vibrations between metal ions (such as Mn^2+^ or Co^3+^) and oxygen ions in spinel-type oxides [24]. In addition, the peaks at 471 cm^−1^ and 607 cm^−1^ are assigned to the octahedral Eg and tetrahedral F_2_g vibrational modes in the spinel-type structure [25]. The peaks at 534.65 cm^−1^ and 1099.69 cm^−1^, with the latter likely associated with the biphonon vibration characteristics of the Mn-Co oxide [26]. The above results indicate the presence of cationic defects in Mn_0.5_Co_2.5_O_4_, with the chemical environment of Mn and Co differing from the stoichiometric MnCo_2_O_4_, further confirming the successful synthesis of Mn_0.5_Co_2.5_O_4_. After Ni doping, the sample exhibits significant peak shifts and peak broadening in the 100–200 cm^−1^ and 400–900 cm^−1^ regions compared to the undoped sample, which may be related to the substitution of Co^2+^ atoms by Ni and the resulting lattice distortion and defects [27]. These phenomena suggest that Ni doping may induce lattice disorder in the Mn_0.5_Co_2.5_O_4_ sample, leading to structural defects and further affecting the excitation of phonon modes.

The XPS survey spectra (Figure 1b) confirm the coexistence of Mn, Co, and O elements in Mn_0.5_Co_2.5_O_4_. In addition to these elements, the spectrum of Ni−Mn_0.5_Co_2.5_O_4_ also shows the presence of Ni, confirming the successful incorporation of Ni. The Ni 2p XPS high-resolution spectrum of Mn_0.5_Co_2.5_O_4_ (Figure 1c) shows no significant peaks; however, the distinct Ni 2p peaks in Ni−Mn_0.5_Co_2.5_O_4_ confirm the successful Ni incorporation, with two broad satellite peaks and two main peaks, deconvoluted to show the presence of Ni^2+^ and Ni^3+^ oxidation states [28]. The Mn 2p spectrum of Mn_0.5_Co_2.5_O_4_ (Figure 1d) splits into Mn 2p_1/2_ and Mn 2p_3/2_ peaks, with deconvolution indicating that the main oxidation states of Mn are Mn^2+^ and Mn^3+^ [11,29,30,31,32]. Noticeably, compared to pure-phase MnCo_2_O_4_ [33], the Mn 2p peak of Mn_0.5_Co_2.5_O_4_ shifts to lower binding energy, which is attributed to changes in the chemical environment of Mn, indicating the successful synthesis of Mn_0.5_Co_2.5_O_4_. In Ni−Mn_0.5_Co_2.5_O_4_, the Mn 2p_1/2_ and Mn 2p_3/2_ peaks shift to higher binding energy, suggesting successful Ni incorporation and competitive substitution interactions with Mn. Additionally, unlike Mn_0.5_Co_2.5_O_4_, the main oxidation states of Mn after Ni doping are Mn^2+^, Mn^3+^, and Mn^4+^, with the presence of Mn^4+^ likely due to the oxidation effect of Ni^2+^, which increases the oxidation state of Mn [34]. The Co 2p spectrum of Mn_0.5_Co_2.5_O_4_ (Figure 1e) can be fitted into Co 2p_1/2_ and Co 2p_3/2_ peaks. Compared to MnCo_2_O_4_, the shift of the Co 2p_3/2_ peak in Mn_0.5_Co_2.5_O_4_ to lower binding energy indicates a change in the energy gap of Co, further confirming the successful synthesis of Mn_0.5_Co_2.5_O_4_ [33,35]. After deconvolution, the oxidation states of Co in Mn_0.5_Co_2.5_O_4_ are predominantly Co^2+^ and Co^3+^ [2,36,37]. Compared to Mn_0.5_Co_2.5_O_4_, although the binding energy of Co in Ni−Mn_0.5_Co_2.5_O_4_ did not show a significant shift, it is important to note that analysis reveals an increase in the Co^3+^/Co^2+^ ratio. The O 1s spectrum (Figure 1f) splits into three peaks: lattice oxygen (O1), adsorbed oxygen (O2), and surface-absorbed water molecules (O3) [1,34]. After Ni doping, except for a slight rightward shift of the O2 peak, the other peaks showed no significant shifts. Additionally, a significant decrease in the O3 peak was clearly observed. This may be attributed to the structural reconstruction or lattice distortion induced by doping. The structural changes could lead to the desorption of water originally adsorbed on the surface. When the doping ions enter the lattice and cause lattice expansion or contraction, the surface structure where the adsorbed water resides becomes unstable, making it easier for the adsorbed water to desorb, which results in the observed decrease of the O3 peak in the XPS spectra [38]. The 2p orbital peaks of Mn and Co, the binding energies of the main peaks, and the normalized oxidation states of each metal element are summarized (see Tables S2 and S3).

According to the doping preference theory, in addition to Mn^2+^ being substituted by Ni^2+^, Ni^2+^ (0.69 Å) may also replace Co^2+^ (0.65 Å), and Ni^3+^ (0.56 Å) could substitute Mn^3+^ (0.58 Å) [39]. Based on the elemental compositions and valence ratios obtained from ICP-OES and XPS, the chemical formulas of the two samples are as follows: Mn^II^_0.221_Mn^III^_0.279_Co^II^_1.541_Co^III^_0.959_O and Ni^II^_0.029_Ni^III^_0.016_Mn^II^_0.044_Mn^III^_0.058_ Mn^VI^_0.052_Co^II^_1.402_Co^III^_1.004_O_4_ (see Table S4). In summary, Ni doping facilitates the formation of higher-valence Mn^4+^, which enhances multivalent state conversion. Additionally, the increased Co^3+^/Co^2+^ ratio widens the electrochemical window, which contributes to the improved specific capacitance [40].

**Figure 2:**
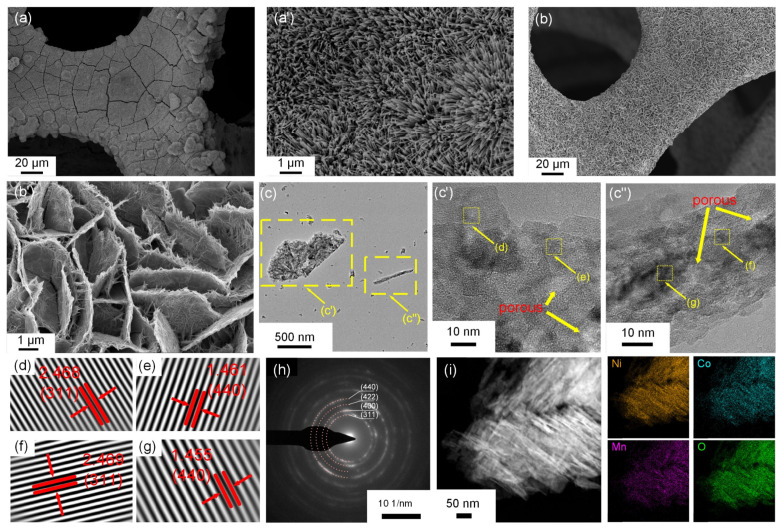
(**a**,**b**) SEM images of Mn_0.5_Co_2.5_O_4_ and Ni−Mn_0.5_Co_2.5_O_4_, along with corresponding high magnification images (**a′**,**b′**). Ni−Mn_0.5_Co_2.5_O_4_: (**c**) HRTEM images and corresponding (**c′**) nanosheets and (**c″**) nanowires, (**d**,**e**) nanoplates lattice fringes, and (**f**,**g**) nanowires lattice fringes; (**h**) SAED image; (**i**) EDS image.

The growth of Ni−Mn_0.5_Co_2.5_O_4_ on nickel foam (Figure 2b) effectively mitigates the cracking, overlapping, and agglomeration issues observed in Mn_0.5_Co_2.5_O_4_ (Figure 2a), which enhances electrolyte exposure, improves specific capacitance, and reduces diffusion impedance [41,42]. High-magnification images (Figure 2a′,b′) reveal that Mn_0.5_Co_2.5_O_4_ exhibits a nanowire morphology, while the morphology of Ni−Mn_0.5_Co_2.5_O_4_ transforms into a cross-linked nanoflake–nanowire structure with a 5 μm edge length and 200 nm thickness upon Ni doping. The TEM image (Figure 2c) shows a rough nanoflake–nanowire structure, which is consistent with the SEM results. The HRTEM image of the nanoflake (Figure 2c′) shows well-defined lattice fringes, with a lattice spacing of 2.468 Å, slightly larger than the standard PDF card value of 2.462 Å, indicating that Ni doping causes a slight lattice expansion [43]. The nanowire image (Figure 2c″) shows similar structural characteristics. The SAED pattern (Figure 2h) shows d-spacings of 2.467, 2.035, and 1.664 Å corresponding to the (311), (400), and (422) planes, respectively. Notably, while the d-spacing of the (311) plane shows a slight expansion compared to Mn_0.5_Co_2.5_O_4_, no significant changes are observed in other planes, which is consistent with the XRD results and further supports that Ni doping does not affect the lattice stability of this material with significant defects. EDS analysis (Figure 2i) confirms that the Ni, Mn, Co, and O elements are uniformly distributed within the Ni−Mn_0.5_Co_2.5_O_4_ nanoflake–nanowire structure.

### 2.2. Electrochemical Test Results

**Figure 3:**
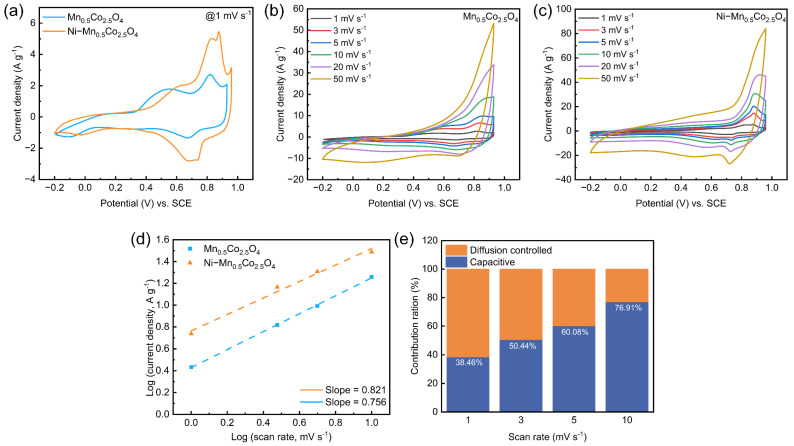
Electrode materials of Mn_0.5_Co_2.5_O_4_ and Ni−Mn_0.5_Co_2.5_O_4_: (**a**) CV curves at 1 mV s^−1^; CV curves at different scan rates for (**b**) Mn_0.5_Co_2.5_O_4_ and (**c**) Ni−Mn_0.5_Co_2.5_O_4_; (**d**) logarithmic plot of peak cathodic current versus scan rate for Mn_0.5_Co_2.5_O_4_; (**e**) proportional histogram of capacitance contributions for Ni−Mn_0.5_Co_2.5_O_4_ from 1 to 10 mV s^−1^.

The CV curve at a scan rate of 1 mV s^−1^ (Figure 3a) shows that non-stoichiometric Mn_0.5_Co_2.5_O_4_ exhibits a wide potential window of 1.13 V, which is superior to the stoichiometric MnCo_2_O_4_ and its composites reported in the literature (Table S5) [16]. Upon Ni doping, the potential window of Ni−Mn_0.5_Co_2.5_O_4_ slightly increases to 1.16 V. Notably, the number of redox peaks in the CV curve increases after Ni doping, which is likely due to the redox exchange between Mn^3+^ and Mn^4+^. Furthermore, the significant enlargement of the area under the CV curve provides further evidence that the rational doping of Ni enhances the specific capacitance of the material [44]. The prominent redox peaks observed in the CV curves indicate the pseudocapacitive behavior of both materials. Additionally, the more symmetric voltage difference further demonstrates that the Ni−Mn_0.5_Co_2.5_O_4_ electrode is less affected by polarization [45]. The b-values calculated from the CV curves at various scan rates (Figure 3b,c) for both samples (Figure 3d) are greater than 0.5 but less than 1, indicating that both materials exhibit characteristics that are governed by a combination of capacitive and battery-like behavior. Additionally, the reaction kinetics of Ni−Mn_0.5_Co_2.5_O_4_ at scan rates ranging from 1 to 10 mV s^−1^ (Figure 3e) were quantified, revealing that the capacitance contribution of the material increased from 38.46% to 76.91%, further demonstrating the fast reaction kinetics of the material.

**Figure 4:**
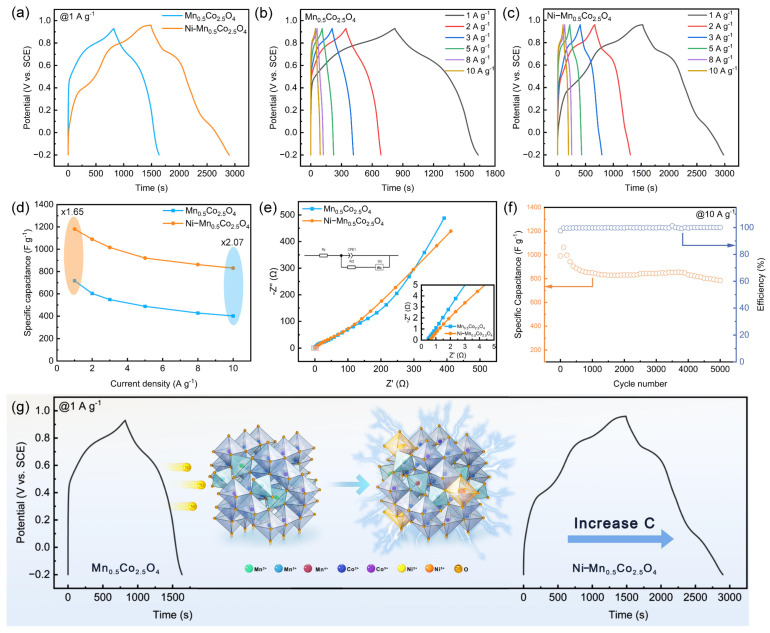
Mn_0.5_Co_2.5_O_4_ and Ni−Mn_0.5_Co_2.5_O_4_: (**a**) GCD curves at a current density of 1 A g^−1^; (**b**,**c**) GCD curves at various current densities; (**d**) specific capacitance curves at each current density; (**e**) Nyquist spectra; (**f**) cycle performance characteristics of Ni−Mn_0.5_Co_2.5_O_4_; (**g**) diagram illustrating the enhancement of Ni−Mn_0.5_Co_2.5_O_4_ electrode via multi-active-site synergistic effect.

The GCD curves at a current density of 1 A g^−1^ for both Mn_0.5_Co_2.5_O_4_ and Ni−Mn_0.5_Co_2.5_O_4_ (Figure 4a), along with the corresponding calculations from the Supporting Information (Equation (S1)), reveal that the specific capacitance of Mn_0.5_Co_2.5_O_4_ reaches 716.9 F g^−1^ at 1 A g^−1^, which is notably higher than that of stoichiometric MnCo_2_O_4_ electrodes and their composites reported in the literature (Table S5). This enhancement highlights the favorable role of lattice defects in improving the specific capacitance of the electrode material. Subsequently, analysis of the GCD curve for Ni−Mn_0.5_Co_2.5_O_4_ reveals several inflection points that align well with the CV curve. Additionally, the significantly increased discharge time indicates that its specific capacitance surpasses that of Mn_0.5_Co_2.5_O_4_. The specific capacitance of Ni−Mn_0.5_Co_2.5_O_4_ at 1 A g^−1^ is calculated to be 1180.6 F g^−1^, which is 64.7% higher than that of Mn_0.5_Co_2.5_O_4_, outperforming MnCo_2_O_4_ electrodes reported in the literature (Table S5). According to previous studies, Co^3+^ in a higher oxidation state is more electrochemically active than Co^2+^ [40], and XPS results show that Ni doping increases the number of Co^3+^ sites, thus promoting the reaction kinetics. Moreover, Ni doping diversifies the oxidation states of Mn, creating Mn^4+^/Mn^3+^ and Mn^3+^/Mn^2+^ redox couples that enhance electron transfer capabilities [46]. The specific capacitance curves for both samples at various current densities (Figure 4d) are derived from the GCD curves shown in Figure 4b,c, with capacitance retention at 10 A g^−1^ increasing from 56.1% to 70.4%. The key capacitance performance parameters of the two samples are summarized in Table S6.

The Nyquist plots, the fitted equivalent circuit diagrams (Figure 4e), and the impedance fitting parameters (detailed in Table S7) of the Mn_0.5_Co_2.5_O_4_ and Ni−Mn_0.5_Co_2.5_O_4_ samples were analyzed. In the high-frequency region (Figure 4e inset), it was observed that both samples show extremely low solution contact resistance (Rs). However, neither sample exhibits a distinct and complete semicircular arc in the high-frequency part, which suggests that the behavior of the electrodes may primarily be controlled by capacitive reactions, rather than dominated by charge transfer processes. This is consistent with the calculated results in Figure 3d, indicating the rapid nature of the charge transfer process, which is closely related to the high conductivity of the materials and the enhanced interfacial reaction kinetics [47]. Further analysis indicates that Ni doping significantly reduces the charge transfer resistance (Rct) of Mn_0.5_Co_2.5_O_4_ from 109.26 Ω to just 2.627 Ω in Ni−Mn_0.5_Co_2.5_O_4_, a decrease by 41.6 times. The electronic properties of the samples were probed using ultraviolet-visible absorption spectra (Figure S2a), and the optical band gaps of the samples were estimated using the Tauc equation (Figure S2b) [48,49]. The analysis shows that after Ni doping into the Mn_0.5_Co_2.5_O_4_ structure, the optical band gap reduced from 1.01 eV to 0.84 eV, which creates more favorable conditions for the excitation of charge carriers to the conduction band, lowers the energy barrier for electronic transitions, and facilitates the movement of more charge carriers into the conduction band, thus improving the electrical conductivity of Mn_0.5_Co_2.5_O_4_ [50].

In addition, the *p*-values of the constant phase element (CPE1) for the two samples, as given in Table S7, were fitted to 0.594 and 0.676, respectively. Generally, when *p* = 1, the CPE behaves as an ideal capacitor, while when *p* = 0, the CPE behaves as an ideal resistor. When the *p*-value is between 0 and 1, the closer *p* is to 1, the more the interface approaches ideal capacitive behavior. Conversely, the smaller the *p*-value, the more the system deviates from ideal capacitance, exhibiting more non-ideal characteristics [51,52]. The *p*-value for Ni−Mn_0.5_Co_2.5_O_4_ is 0.676, indicating that the system’s capacitive behavior is closer to ideal capacitance [53]. Specifically, slight inhomogeneity or local structural variations may exist on the electrode surface, potentially related to factors such as surface roughness, defects, or local variations in current density. However, compared to Mn_0.5_Co_2.5_O_4_ with a lower *p*-value, despite the presence of some inhomogeneity, this electrode material demonstrates better capacitive performance and a faster electrochemical reaction rate. Overall, Ni−Mn_0.5_Co_2.5_O_4_ exhibits a relatively uniform surface structure and an efficient charge transfer process.

In summary, the incorporation of Ni markedly enhances charge transfer reactions and augments electron transport capabilities. Additionally, Ni doping is likely to bolster pseudocapacitive behavior at the electrode surface, thus optimizing charge transfer efficiency [54]. These substantial improvements are attributed to Ni doping’s dual role in chemically and structurally optimizing the material. Firstly, Ni serves as a p-type dopant, effectively lowering the activation energy and narrowing the band gap, which in turn boosts the conductivity of Ni−Mn_0.5_Co_2.5_O_4_ [55,56]. Secondly, the substitution of larger Ni^2+^ ions for smaller Mn^2+^ ions results in lattice distortions. These distortions induce local expansions or contractions, not only facilitating the opening of ion transport channels but also significantly diminishing diffusion resistance, thereby promoting reaction kinetics [57].

The synergistic effect of multiple active sites plays a crucial role in optimizing the electrochemical performance of the material (Figure 4g). Ni doping promotes the high oxidation state evolution of Mn ions and increases the quantity of highly active Co^3+^ ions, forming multiple active sites within the electrode material. The synergistic effect between these sites significantly enhances the charge storage and transfer capabilities. Through the synergistic effect of multiple active sites, the electrochemical reactivity and ion diffusion ability of the material are significantly improved, ultimately driving the optimization of the overall electrode performance.

Ni−Mn_0.5_Co_2.5_O_4_ demonstrates relatively stable cycling performance, maintaining 79.2% capacity retention after 5000 cycles at 10 A g^−1^, with an outstanding coulombic efficiency of 100.1% (Figure 4f). These results fully demonstrate the superior performance of the phase-deficient spinel Mn_0.5_Co_2.5_O_4_ and the further optimization achieved through microwave-assisted Ni doping.

**Figure 5:**
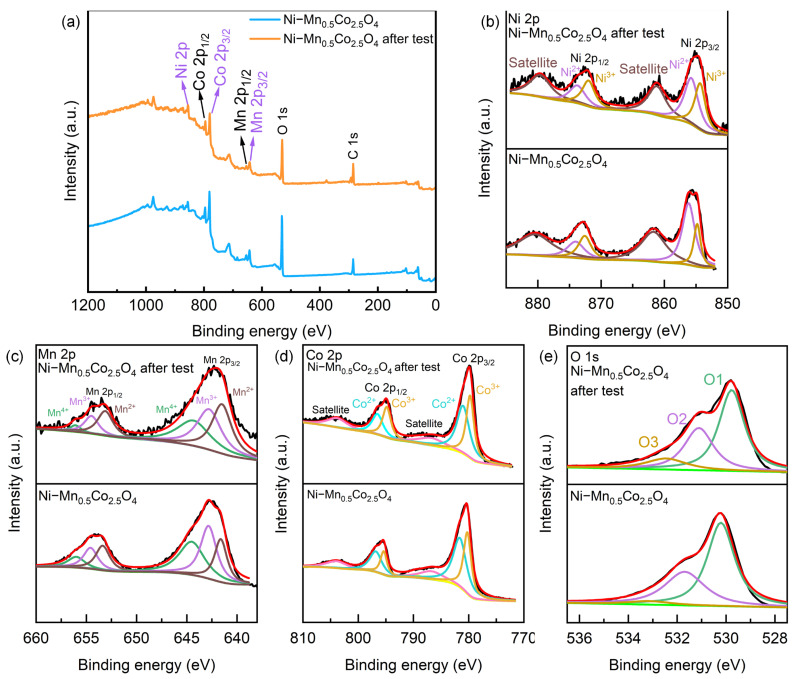
Ni−Mn_0.5_Co_2.5_O_4_ before and after cycling tests: (**a**) XPS spectrum, (**b**) Ni 2p, (**c**) Mn 2p, (**d**) Co 2p, and (**e**) O 1s.

XPS analysis revealed the surface composition changes of Ni−Mn_0.5_Co_2.5_O_4_ after cycling tests. The XPS survey spectrum results indicate no significant change in the material’s overall chemical components. However, fine spectra analysis (Figure 5a) shows that the oxygen spectrum shifts towards lower binding energies, suggesting that the material’s surface may have undergone a reduction process [58]. This change is closely related to the decrease in charge transfer efficiency and the decline in specific capacitance. By calculating the elemental composition ratios after 5000 cycles of constant current charge–discharge tests through the fine spectra of each element and analysis (see Table S8 in the Supporting Information), the proportion of Co^3+^ significantly decreased, while the proportion of Co^2+^ increased, leading to a reduction in highly active sites and an increase in low-active sites, thereby inhibiting the electrode’s redox activity. Although the increase in Ni^3+^ replaces Mn^3+^ to enhance the redox reaction capability, the decrease in Ni^2+^ and its tendency to replace the low-activity Mn^2+^ limit the improvement of charge storage and release capabilities. At the same time, the increase in Mn^2+^ and the decrease in Mn^3+^ further reduce the number of high-active sites, slowing down the electrochemical reaction rate and affecting the retention of specific capacitance. Overall, although the increase in Ni^3+^ and Co^3+^ may benefit the redox reactions, their increase could also reduce the long-term stability of the material, thereby affecting conductivity and specific capacitance retention. To comprehensively reveal the microscopic evolution of the material during cycling, in addition to changes in chemical components, Figure S3 provides high- and low-magnification SEM images of Ni−Mn_0.5_Co_2.5_O_4_ after cycling, visually demonstrating the morphological evolution of the material. The results show that after 5000 cycles, the nanosheet-wire structure of Ni−Mn_0.5_Co_2.5_O_4_ slightly collapsed compared to the uncycled electrode, indicating that the material underwent structural degradation during the electrochemical reaction process. This morphological change is closely related to changes in the surface chemical components and may have further exacerbated the decline in charge transfer efficiency and the deterioration of specific capacitance. Therefore, both surface chemical components changes and morphological evolution jointly led to the reduced cycling stability of the doped material compared to the undoped Mn_0.5_Co_2.5_O_4_. Based on this finding, future research should focus on improving the material’s structural integrity and surface stability through optimized synthesis processes, designing stable structures, and introducing surface protection strategies, so as to effectively extend its cycling life while maintaining good specific capacitance and rate performance, thus improving long-term electrochemical performance [59,60].

## 3. Materials and Methods

All chemicals were of analytical grade and used without further purification. Cobalt nitrate hexahydrate (Co(NO_3_)_2_·6H_2_O), manganese chloride tetrahydrate (MnCl_2_·4H_2_O), and nickel sulfate hexahydrate (NiSO_4_·6H_2_O) were purchased from Aladdin Industrial Corporation, Shanghai, China. Ammonium fluoride (NH_4_F) was purchased from Shanghai Macklin Biochemical Co., Ltd., Shanghai, China. Urea ((NH_2_)_2_CO) was purchased from Guangdong Guanghua Chemical Factory Co., Ltd., Shantou, China. The microwave hydrothermal synthesis apparatus was purchased from Beijing Xianghu Technology Development Co., Ltd., Beijing, China. The tube furnace was purchased from Kemi Instruments, Hefei, China. The electrochemical workstation used is detailed in the electrochemical test section of the Supporting Information.

**Figure 6:**
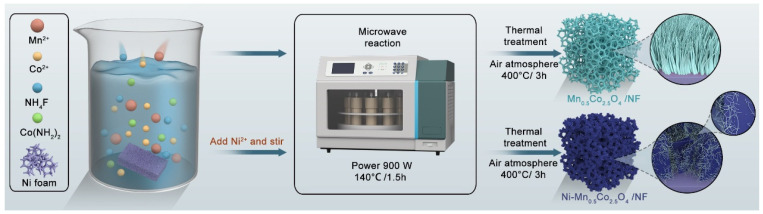
Microwave-assisted synthesis method for preparation of Mn_0.5_Co_2.5_O_4_ and Ni−Mn_0.5_Co_2.5_O_4_ nanowires and nanosheet-nanowire hierarchical structures.

As shown in Figure 6, 0.5 mmol of MnCl_2_·4H_2_O, 1 mmol of Co(NO_3_)_2_·6H_2_O, and appropriate amounts of (NH_2_)_2_CO and NH_4_F were fully dissolved in 30 mL of deionized (DI) water. The pretreated nickel foam was then added, and the above mixture was placed in a microwave-assisted synthesis apparatus, where it was maintained at 140 °C for 1.5 h. The resulting product was then washed and annealed, yielding Mn_0.5_Co_2.5_O_4_ nanowires with significant lattice defects grown in situ on the nickel foam.

Additionally, during the preparation of the above solution, the amount of MnCl_2_·4H_2_O was changed to 0.375 mmol, and 0.125 mmol of NiSO_4_·6H_2_O was added and thoroughly stirred. The rest of the procedure remained unchanged, resulting in the formation of Ni−Mn_0.5_Co_2.5_O_4_ nanosheet-wire hierarchical structures.

The detailed experimental procedure, essential physical characterizations, and electrochemical evaluation processes are provided in the Supporting Information [11,14,61].

## 4. Conclusions

In this study, we synthesized a non-stoichiometric bimetallic oxide material, Mn_0.5_Co_2.5_O_4_, using a microwave-assisted process. Building upon this, we successfully prepared a Ni-doped non-stoichiometric spinel material, Ni−Mn_0.5_Co_2.5_O_4_, featuring a 1D/2D morphology. The results show that under microwave-assisted processing, the spinel structure of the nickel-doped sample remained stable, while its microstructure evolved into a hybrid nanoflake–nanowire hierarchical structure. More importantly, Ni doping induced a transition in the oxidation states of Mn, from Mn^2+/3+^ to a mixed high-valence state of Mn^2+/3+/4+^, and significantly increased the proportion of active Co^3+^ sites. This transformation led to an enhancement of active reaction sites, greatly promoted synergistic effects, and substantially improved the conductivity and energy storage performance of the material. Compared to Mn_0.5_Co_2.5_O_4_, Ni−Mn_0.5_Co_2.5_O_4_ exhibited a 67% increase in specific capacitance (1180.6 F g^−1^ at 1 A g^−1^), a 14.3% improvement in rate performance (831.1 F g^−1^ at 10 A g^−1^), and a 41.6-fold reduction in charge transfer resistance (Rct = 2.627 Ω). The potential window reached 1.16 V, and after 5000 cycles, the material maintained 79.2% of its capacitance with a Coulombic efficiency close to 100%.

## Data Availability

The original contributions presented in this study are included in the article/Supplementary Materials. Further inquiries can be directed to the corresponding authors.

## Supplementary Materials

The following supporting information can be downloaded at: https://www.mdpi.com/article/10.3390/molecules30040873/s1, Figure S1. Raman spectra of Mn_0.5_Co_2.5_O_4_ and Ni−Mn_0.5_Co_2.5_O_4_. Figure S2. UV–vis absorption spectra for Mn_0.5_Co_2.5_O_4_ and Ni−Mn_0.5_Co_2.5_O_4_. Figure S3. After 5000 cycles of constant current charge–discharge testing, high-and low-magnification SEM images of Ni−Mn_0.5_Co_2.5_O_4_. Table S1. Crystallite sizes (D), d-spacing (d), and lattice constant of Mn_0.5_Co_2.5_O_4_ and Ni−Mn_0.5_Co_2.5_O_4_ nanoparticles. Table S2. 2p orbital peak binding energy positions of elements Mn and Co. Table S3. Values of main peak binding energies of elements Ni, Mn, Co, and O. Table S4. Ratio relation of different Mn_0.5_Co_2.5_O_4_ and Ni−Mn_0.5_Co_2.5_O_4_ samples evaluated using ICP-OES and XPS. Table S5. Electrochemical properties of previously reported MnCo_2_O_4_ electrode materials. Table S6. Optimized comparison of energy densities of Mn_0.5_Co_2.5_O_4_ and Ni−Mn_0.5_Co_2.5_O_4_. Table S7. EIS fitting parameters for Mn_0.5_Co_2.5_O_4_ and Ni−Mn_0.5_Co_2.5_O_4_. Table. S8. Table of ion composition ratios of Ni−Mn_0.5_Co_2.5_O_4_ calculated by XPS before and after cycling tests [62,63,64,65,66,67,68,69,70,71,72,73].
