# Supplementary material for: Microwave-Assisted Doping Engineering Construction of Spinel-Structured Nonstoichiometric Manganese Cobaltite with Mixed 1D/2D Morphology for Supercapacitor Application
Source: Molecules. 2025 Feb 14;30(4):873. doi: 10.3390/molecules30040873 (PMC11858410; doi:10.3390/molecules30040873)
Supplement: Supplementary file 1 [file molecules-30-00873-s001.zip › molecules-3350813-supplementary.pdf]

Supplementary Information for

Microwave-Assisted Doping Engineering Construction of Spinel-Structured Nonstoichiometric Manganese Cobaltite with Mixed 1D/2D Morphology for Supercapacitor Application

Yuxuan Sheng [1,2,3], Yin Sun [1,2], Jin Yan [1,2,3,*], Wei Wang [1,2], Shuhuang Tan [1,2], Yuchen Lin [1,2], Haowei Wang [1,2], Yichen Liu [1,2], Baotong Xie [1,2] and Xiaoran Sun [1,2]

[1] Naval Architecture and Shipping College, Guangdong Ocean University, Zhanjiang 524088, China
[2] Guangdong Provincial Key Laboratory of Intelligent Equipment for South China Sea Marine Ranching, Guangdong Ocean University, Zhanjiang 524088, China
[3] School of Mechanical Engineering, Guangdong Ocean University, Zhanjiang 524088, China
* Correspondence: yanj@gdou.edu.cn

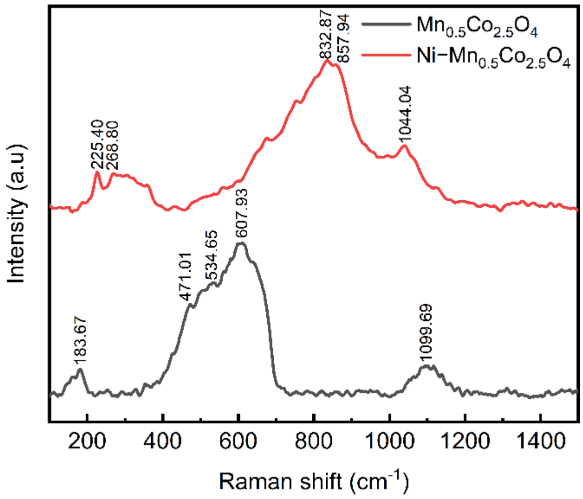

Figure S1. The Raman spectra of $Mn_{0.5}Co_{2.5}O_4$ and Ni−$Mn_{0.5}Co_{2.5}O_4$.

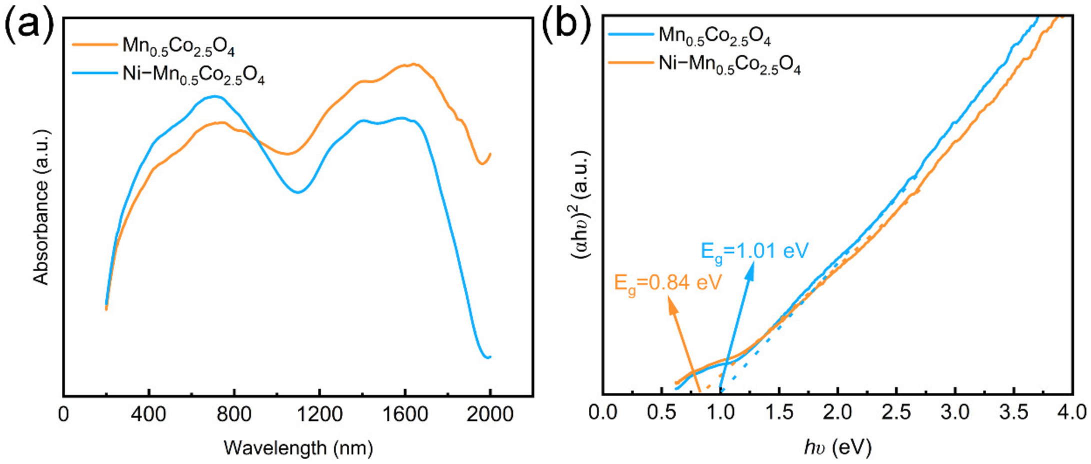

Figure S2. The UV–vis absorption spectra for $Mn_{0.5}Co_{2.5}O_4$ and Ni−$Mn_{0.5}Co_{2.5}O_4$.

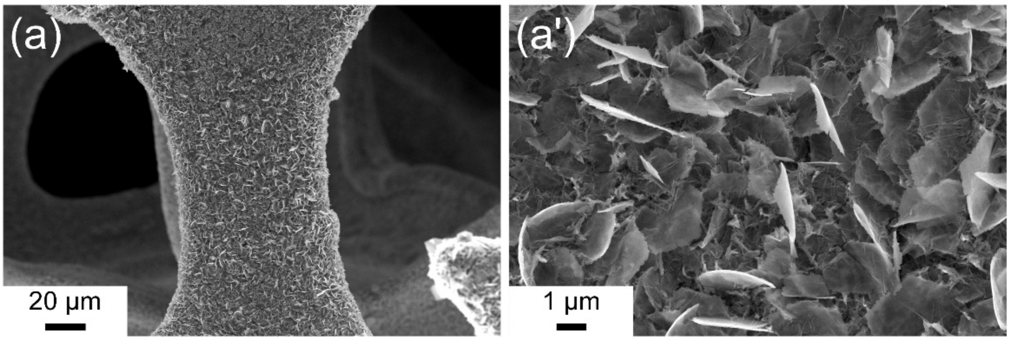

Figure S3. After 5000 cycles of constant current charge-discharge testing, the high-and low-magnification SEM images of Ni−$Mn_{0.5}Co_{2.5}O_4$.

1. Experiment

1.1 Synthesis of Deficient Spinel $Mn_{0.5}Co_{2.5}O_4$ and Ni-doped Deficient Spinel Ni−$Mn_{0.5}Co_{2.5}O_4$ with Significant Defects Loaded on Nickel Foam via Microwave-Assisted Method

$Mn_{0.5}Co_{2.5}O_4$ with substantial lattice defects and its Ni-doped derivative, Ni −$Mn_{0.5}Co_{2.5}O_4$, were synthesized via microwave-assisted hydrothermal technique. As depicted in Figure 5, nickel foam (NF) was first cut into 1×3 cm^2 sections, then cleaned and air-dried. For the synthesis of $Mn_{0.5}Co_{2.5}O_4$, a solution was prepared by dissolving 1 mmol of $Co(NO_3)_2 \cdot 6H_2O$, 2 mmol of NH_4F, 6 mmol of $(NH_2)_2CO$, and 0.5 mmol of $MnCl_2 \cdot 4H_2O$ in 30 ml of deionized water. This solution was stirred until clear, and then transferred into a polytetrafluoroethylene-lined autoclave that had been loaded with pre-treated nickel foam. The loaded autoclave was placed within a microwave synthesizer, set to 140°C, and maintained at this temperature for 1.5 hours with a power setting of 900 W. After the reaction, the precursor material was removed, thoroughly rinsed, and dried. It was then annealed in a tube furnace at 400°C for 3 hours to obtain $Mn_{0.5}Co_{2.5}O_4$. For the Ni −$Mn_{0.5}Co_{2.5}O_4$ sample, in the above clarification solution, change the amount of $MnCl_2 \cdot 4H_2O$ to 0.375 mmol and add 0.125 mmol of $NiSO_4 \cdot 6H_2O$, with the rest of the process remaining unchanged.

1.2. Materials characterization

XRD (X-ray diffraction) analysis was performed using a BRUKER D8 Advance with Cu Kα radiation covering a 2θ range of 10–90° at a scan rate of 5° min^{-1}. This technique was used to characterize the crystal structures of non-stoichiometric $Mn_{0.5}Co_{2.5}O_4$ and significantly defective Ni −$Mn_{0.5}Co_{2.5}O_4$. XPS (X-ray photoelectron spectroscopy) with a ThermoFischer ESCALAB 250Xi, utilizing an Al Kα radiation source (hv = 1486.6 eV) in a chamber vacuum of $8x10^{-10}$ Pa, analyzed the elemental composition and oxidation states of the synthesized products. SEM (scanning electron microscopy) with a TESCAN MIRA LMS and TEM (transmission electron microscopy) with an FEI Tecnai F20 documented the microstructures and morphologies of the products. ICP-AES (inductively coupled plasma atomic emission spectroscopy) analysis with an ICPS-7500 determined the chemical compositions and concentrations of metal ions in the samples. The ultraviolet−visible spectrum was measured using a Shimadzu UV3600i−PLUS spectrometer.

1.3. Electrochemical measurement

Electrochemical characterisation was carried out using a typical three-electrode system with 6M KOH solution as the electrolyte, a CORRTEST CS2350M electrochemical workstation, a saturated calomel electrode (SCE) as the reference electrode, and a platinum mesh electrode as the counter electrode. The working electrode was a foam nickel electrode cut into 1x1 cm^2, loaded with $Mn_{0.5}Co_{2.5}O_4$ and Ni−$Mn_{0.5}Co_{2.5}O_4$. The specific capacity was determined using the GCD method

(Eq. (S1)). Additionally, cyclic voltammetry (CV) and electrochemical impedance spectroscopy (EIS) were also employed for measurements[11, 14, 61].

$$C_{sp} = (F\ g^{-1}) = \frac{I \cdot \Delta t}{m \cdot \Delta V} \tag{S1}$$

In the formula, I denotes the current during the discharge process; m refers to the mass of the active material loaded onto the nickel foam; Δt represents the time of discharge; ΔV indicates the voltage (V).

1.4. Quantitative Analysis of X-Ray Diffraction Data

XRD analysis involved calculating lattice parameters (D), interplanar spacings (d), and unit cell constants using Scherrer's equation (Eq. (S2)) and Bragg's law (Eq. (S3, S4)).

$$D = \frac{k\lambda}{\beta cos\theta} \tag{S2}$$

$$2dsin\theta = n\lambda \tag{S3}$$

$$\frac{1}{d_{hkl}} = \frac{\sqrt{h^2+k^2+l^2}}{a} \tag{S4}$$

Here, D denotes the average crystallite size, while k represents the Scherrer constant with a value of 0.89. λ indicates the X-ray wavelength, and β corresponds to the full width at half maximum (FWHM) of the X-ray peak observed in the sample. θ refers to the Bragg diffraction angle. d stands for the interplanar spacing, n is the order of diffraction, and d_{hkl} denotes the specific interplanar spacing corresponding to the Miller indices hkl.

Table S1. The crystallite sizes (D), d-spacing (d) and lattice constant of $Mn_{0.5}Co_{2.5}O_4$ and Ni-$Mn_{0.5}Co_{2.5}O_4$ nanoparticles.

**Samples**	**Lattice parameters**				
	a= b= c (Å)	α=β=γ (°)	V (Å^3)	d-spacing value(Å)	crystallite size (nm)
$Mn_{0.5}Co_{2.5}O_4$	8.168	90	544.94	2.463	13.266
Ni −$Mn_{0.5}Co_{2.5}O_4$	8.185	90	548.31	2.468	15.068

Table S2. 2p orbital peak binding energy positions of the elements Mn and Co.

Samples	Mn $2p_{3/2}$ (eV)	Mn $2p_{1/2}$ (eV)	Co $2p_{3/2}$ (eV)	Co $2p_{1/2}$ (eV)
$MnCo_2O_4$	642.4	654.2	780.5	795.6
$Mn_{0.5}Co_{2.5}O_4$	642.0	653.5	780.2	795.6
Ni−$Mn_{0.5}Co_{2.5}O_4$	642.6	653.9	780.4	795.7

Table S3. The values of the main peak binding energies of the elements Ni, Mn, Co, O.

Samples	Ni^{2+} (eV)	Ni^{3+} (eV)	Mn^{2+} (eV)	Mn^{3+} (eV)	Mn^{4+} (eV)	Co^{2+} (eV)	Co^{3+} (eV)	O1 (eV)	O2 (eV)	O3 (eV)
$Mn_{0.5}Co_{2.5}O_4$	N/A	N/A	653.2	654.8	N/A	796.8	795.4	530.0	531.2	532.3
	N/A	N/A	641.8	643.4	N/A	781.5	780.1	N/A	N/A	N/A
Ni−$Mn_{0.5}Co_{2.5}O_4$	874.1	872.6	653.3	654.5	655.9	796.9	795.4	530.2	531.6	533.2
	856.2	854.8	641.6	642.8	644.5	781.6	780.3	N/A	N/A	N/A

Table S4. The ratio relation of different $Mn_{0.5}Co_{2.5}O_4$,Ni−$Mn_{0.5}Co_{2.5}O_4$ samples evaluated using ICP-OES and XPS.

**Atomic ratio**	**Sample**	
	**$Mn_{0.5}Co_{2.5}O_4$**	**Ni−$Mn_{0.5}Co_{2.5}O_4$**
Ni/Co	N/A	0.056
Mn/Co	0.580	0.191
Ni^{2+}/ Ni^{3+}	N/A	1.809
Co^{2+}/Co^{3+}	1.607	1.396
Mn^{2+}/Mn^{3+}	0.792	0.771
Mn^{3+}/Mn^{4+}	N/A	1.126
Molecular formula	$Mn_{0.5}Co_{2.5}O_4$	$Ni_{0.135}Mn_{0.459}Co_{2.406}O_4$
Stoichiometric ratio	$Mn^{II}_{0.221}Mn^{III}_{0.279}Co^{II}_{1.541}Co^{III}_{0.959}O_4$	$Ni^{II}_{0.029}Ni^{III}_{0.016}Mn^{II}_{0.044}Mn^{III}_{0.058}$-$Mn^{VI}_{0.052}Co^{II}_{1.402}Co^{III}_{1.004}O_4$

Table S5. The electrochemical properties of the previously reported $MnCo_2O_4$ electrode material have been reported.

Structure of $MnCo_2O_4$ based material	Operating voltage window	Specific capacitance	Retention	Electrolyte	Methods	Reference
$MnCo_2O_{4.5}$-NWs@NF	0.55 V (0~0.55 V vs. Hg/HgO)	288.47 C g^{-1}/524.5 F g^{-1} (1 A g^{-1})	97.42% (6000 cycles @ 6A g^{-1}) $MnCo_2O_{4.5}$-films@NF//AC HSC	2 M KOH	Hydrothermal and calcination	[62]
$MnCo_2O_4$ HSs/NF	1.0 V (0~1.0 V vs. Hg/HgO)	648.4 C g^{-1}/648.4 F g^{-1} (2 mV s^{-1})	94% (10000 cycles @ 10 A g^{-1})	1M Na_2SO_4	N/A	[63]
$MnCo_2O_4$-discs	0.45 V (0~0.45 V vs SCE)	296.1 C g^{-1}/658 F g^{-1} (1 A g^{-1})	98.63% (5000 cycles @ 6 A g^{-1}) $MnCo_2O_4$-discs//AC ASC	2 M KOH	Hydrothermal and calcination	[64]
$MnCo_2O_{4.5}$ NSs	0.55 V (0~0.55 V vs. Hg/HgO)	304.37 C g^{-1}/553.4 F g^{-1} (1 A g^{-1})	100.04%(5000 cycles @ 6 A g^{-1}) $MnCo_2O_{4.5}$ NSs//AC HSC	2 M KOH	Solvothermal and calcination	[65]
$Co_{1.91}Mn_{1.09}O_4$	0.45V (0~0.45 V vs SCE)	146.7 C g^{-1}/326 F g^{-1} (1A g^{-1})	83.3% (10000 cycles @ 2 A g^{-1})	6 M KOH	Hydrothermal and calcination	[66]
$MnCo_2O_4$ microspheres	1.0 V (0~1.0 V vs. SCE)	160.2 C g^{-1}/160.2 F g^{-1} (1A g^{-1})	97% (3000 cycles @ 3 A g^{-1})	6 M KOH	Hydrothermal and calcination	[67]
$MnCo_2O_4$	0.7 V (-0.2~0.5 V vs Ag/AgCl)	133.1 C g^{-1}/190.2 F g^{-1} (2 mV s^{-1})	N/A	6 M KOH	Indirect microwave heating and calcination	[68]
$MnCo_2O_4$ microspheres	0.4 V (0–0.4 V vs Ag)	175.6 C g^{-1}/439 F g^{-1} (1 A g^{-1})	80.6 % (10000 cycles @ 1 A g^{-1})	1 M KOH	Solvothermal and calcination	[69]
Ni-doped $MnCo_2O_4$	1.4 V (-1~0.4 V vs. Ag/AgCl)	529.2 C g^{-1}/378 F g-1 (1 A g^{-1})	84 % (1000 cycles @ 2 A g^{-1})	6 M KOH	Solvothermal and calcination	[16]

$MnCo_2O_4$ nanoflakes	0.4 V (0.1~0.5 V vs. SCE)	86.6 C g^{-1}/216.5 F g^{-1} (0.5 A g^{-1})	96 % (10000 cycles @ 5 A g^{-1})	3 M KOH	Microwave-hydrothermal and calcination	[70]
CF@$Co_{1.5}Mn_{1.5}O_4$	0.5 V (-0.0~0.5 V vs SCE)	275 C g^{-1}/550 F g^{-1} (1 A g^{-1})	97 % (1000 cycles @ 10 mV s^{-1})	1 M KOH	Electrospinning	[71]
$MnCo_2O_4$	1.3 V (-1.0–0.3 V vs SCE)	381.3 C g^{-1}/495.6 F g^{-1} (2 mV s^{-1})	33.05 % (5000 cycles @ 5 A g^{-1})	1 M KOH	Hydrothermal and calcination	[72]
$MnCo_2O_4$	1.0 V (0.0~1.0 V vs SCE)	280 C g^{-1}/280 F g^{-1} (1 A g^{-1})	99 % (10000 cycles @ 5 A g^{-1})	2 M KOH	Hydrothermal and calcination	[73]
**$Mn_{0.5}Co_{2.5}O_4$**	**1.13 V (-0.2~0.93 V vs SCE)**	**810.1 C g^{-1}/716.9 F g^{-1} (1 A g^{-1})**	**77.9 % (12000 cycles @ 10 A g^{-1})**	**6 M KOH**	**Microwave-hydrothermal and calcination**	**This work**

Table S6. Optimized comparison of the energy densities of $Mn_{0.5}Co_{2.5}O_4$ and Ni−$Mn_{0.5}Co_{2.5}O_4$.

**Sample names**	**Operating voltage window （V）**	**Specific capacitance （F g^{-1}）**	**rate of ascension**	**rate capability (10 A g^{-1})**
$Mn_{0.5}Co_{2.5}O_4$	1.13 (-0.2~0.93 vs. SCE)	716.9	N/A	56.1%
Ni−$Mn_{0.5}Co_{2.5}O_4$	1.16 V (-0.2~0.96 vs. SCE)	1180.6	64.7%	70.4%

Table S7. EIS fitting parameters for $Mn_{0.5}Co_{2.5}O_4$ and Ni−$Mn_{0.5}Co_{2.5}O_4$.

**Element**	$Mn_{0.5}Co_{2.5}O_4$	Ni−$Mn_{0.5}Co_{2.5}O_4$
**Rs (Ω)**	0.485	0.592
**CPE1-T**	0.004332	0.0010836
**CPE1-P**	0.59735	0.67617
**Rct**	109.26	2.627
**Wo-R**	554.19	301.57
**Wo-T**	35.541	4.716
**Wo-P**	0.36653	0.28896

Table. S8 Table of ion composition ratios of Ni−$Mn_{0.5}Co_{2.5}O_4$ calculated by XPS before and after cycling tests.

**Atomic ratio**	**Sample**	
	**Ni−$Mn_{0.5}Co_{2.5}O_4$**	**Ni−$Mn_{0.5}Co_{2.5}O_4$ after test**
Ni^{2+}/Ni^{3+}	1.809	1.132 ↓
Co^{2+}/Co^{3+}	1.396	1.599 ↑
Mn^{2+}/Mn^{3+}	0.771	1.110 ↑
Mn^{3+}/Mn^{4+}	1.126	1.360 ↑
